# Proximal Tubular Development Is Impaired with Downregulation of MAPK/ERK Signaling, HIF-1*α*, and Catalase by Hyperoxia Exposure in Neonatal Rats

**DOI:** 10.1155/2019/9219847

**Published:** 2019-08-28

**Authors:** Xuewen Xu, Kai You, Renge Bu

**Affiliations:** ^1^Department of Urology, Shengjing Hospital of China Medical University, Shenyang 110004, China; ^2^Department of Neonatology, Shengjing Hospital of China Medical University, Shenyang 110004, China

## Abstract

Supplemental oxygen therapy (hyperoxia) is a widely used treatment for alveolar hypoxia in preterm infants. Despite being closely monitored, hyperoxia exposure is believed to undermine neonatal nephrogenesis and renal function caused by elevated oxidative stress. Previous studies have mostly focused on the hyperoxia-induced impairment of glomerular development, while the long-term impact of neonatal hyperoxia on tubular development and the regulatory component involved in this process remain to be clarified. Here, we examined tubular histology and apoptosis, along with the expression profile of mitogen-activated protein kinase (MAPK)/extracellular signal-regulated kinase (ERK) signaling, hypoxia-inducible factor 1*α* (HIF-1*α*), and catalase, following hyperoxia exposure in neonatal rats. Hematoxylin and eosin (H&E) staining revealed the early disappearance of the nephrogenic zone, as well as dilated lumens and reduced epithelial cells, of mature proximal tubules following neonatal hyperoxia. A robust increase in tubular cell apoptosis caused by neonatal hyperoxia was found using a TUNEL assay. Moreover, neonatal hyperoxia altered renal MAPK/ERK signaling activity and downregulated the expression of HIF-1*α* and catalase in the proximal tubules throughout nephrogenesis from S-shaped bodies to mature proximal tubules. Cell apoptosis in the proximal tubules was positively correlated with HIF-1*α* expression on the 14th postnatal day. Our data indicates that proximal tubular development is impaired by neonatal hyperoxia, which is accompanied by altered MAPK/ERK signaling as well as downregulated HIF-1*α* and catalase. Therapeutic management that targets MAPK/ERK signaling, HIF-1*α*, or catalase may serve as a protective agent against hyperoxia-induced oxidative damage to neonatal proximal tubules.

## 1. Introduction

The structural and functional development of the neonatal kidney is responsible for postnatal adaptation to extrauterine life [1]. Developmental changes of the kidney after birth involve the further maturation and hypertrophy of nephrons, which continue until adult morphology and size are reached from 3 to 5 years of age. The glomerular filtration rate (GFR) increases with the progression of neonatal renal function, reaching adult levels by 2 years of age [2]. The postnatal maturation of renal tubules follows this rapid maturation and is characterized by a 10-fold increase in proximal tubular length and diameter within the weaning period. The diluting capacity of urine in newborn infants is similar to that of adults. Functional maturity and full urinary concentrating ability are reached at approximately 18 months of age [1]. Low oxygen concentrations (1–3%) are optimal for renal vascular and tubular development in newborn rats [3]. Moreover, an appropriate oxygen tension is critically required in the final papillary development [4].

Since alveolar hypoxia can lead to inadequate tissue oxygenation, pulmonary vasoconstriction, and pulmonary artery hypertension, supplemental oxygen therapy (hyperoxia) is widely used in neonatology and plays a vital role in the management of preterm infants [5]. Despite close monitoring, mounting evidence from various clinical and experimental observations suggests that neonatal hyperoxia causes systemic injury to several organs. Hyperoxia is a key contributor to neonatal and pediatric lung diseases, including airway disease (wheezing and asthma) and bronchopulmonary dysplasia [6]. Chronic exposure to hyperoxia causes oxidative stress and contributes to the pathogenesis of injury in the preterm as well as full-term brain, with a dramatic deterioration of brain function in later life [7]. Neonatal hyperoxia causes severe vitreoretinal pathologic changes that persist into adulthood [8]. Thus, it is important to clarify the adverse impact of neonatal hyperoxia upon nephrogenesis.

Accumulating evidence suggests that neonatal hyperoxia impairs nephrogenesis. Neonatal mice exposed to hyperoxia had smaller glomeruli in both the short and long term [9, 10]. Crescentic glomeruli, which indicate glomerular injury, were markedly increased in 11-month-old, hyperoxia-exposed rats [11]. Hyperoxia led to increases in renal tubular necrosis, dilation, and interstitial inflammation, with higher tubular injury scores [12, 13]. Capillary density per kidney was decreased by neonatal hyperoxia [14]. Impaired renal development is considered a pathophysiological process in that neonatal hyperoxia-induced oxidative stress may initiate apoptosis of renal cells, causing subclinical acute kidney injury or even kidney fibrosis [10, 13, 15, 16]. The hyperoxia-induced early differentiation of renal epithelial cells is another explanation for impaired nephrogenesis [17]. Renal function can also be impaired; for instance, creatinine clearance at 5 months was significantly reduced following the exposure of neonates to hyperoxia [11]. Previous studies on the hyperoxia-induced impairment of nephrogenesis have mainly focused on glomeruli, while the long-term effect of neonatal hyperoxia to tubular development remains unclear.

Several molecular pathways and genes are involved in regulating the complex process of nephrogenesis, such as transcriptional regulators, growth factors, oncogenes, the extracellular matrix, and vascular factors [1, 15]. As a proliferative and antiapoptotic signaling pathway, MAPK/ERK signaling regulates the maintenance and differentiation of kidney progenitor cells in kidney development [18]. Hypoxia-inducible factor 1*α* (HIF-1*α*) is known to be essential for organogenesis by regulating the expression of numerous factors involved in angiogenesis, cellular proliferation, and apoptosis [10]. Catalase plays a critical protective role against oxidative stress in embryogenesis [19]. However, the dynamic expression profile of MAPK/ERK signaling, HIF-1*α*, and catalase in response to neonatal hyperoxia remains undefined. Thus, the aim of this study is to investigate the long-term effect of neonatal hyperoxia on tubular development as well as MAPK/ERK signaling activity, HIF-1*α*, and catalase expression.

## 2. Materials and Methods

### 2.1. Animal Model

Sprague-Dawley (SD) rats were purchased from the Experimental Animal Center, Shengjing Hospital of China Medical University (Shenyang, China). Pregnant SD rats were housed in transparent cages of our laboratory one week before delivery. Pups were delivered naturally when the gestational age was 21 to 23 days. All pups were separated from mothers within 12 h after delivery and were randomly divided into normoxia (fraction of inspired air (FiO_2_) = 0.21, *n* = 80) and hyperoxia groups (FiO_2_ = 0.85, *n* = 80). The inhaled oxygen concentration was measured and recorded continuously with an analyzer equipped with a strip-chart recorder (model 572; Servomex, Norwood, MA, USA). Other environmental conditions of the hyperoxia group were the same as those of the normoxia group except for inhaling room air. Using alkaline lime to absorb CO_2_, the CO_2_ concentration was maintained to less than 0.5%. Room temperature (25~27°C), humidity (60%~70%), and daily light and dark circulation were monitored and regulated automatically. Rats in the hyperoxia group were transited and cross-fostered by mother rats in a normoxia environment every 24 h to avoid the toxicity of hyperoxia and to equalize their nutritional condition. The dams, food, and drinking water were renewed every 24 h. Rats were scheduled for euthanasia in CO_2_ on postnatal days 1, 3, 5, 7, 10, 14, 30 (exposed to normoxia or hyperoxia in the first 14 days), and 60 (exposed to normoxia or hyperoxia in the first 14 days), respectively. The carcasses were dissected and bilateral kidneys were obtained immediately after euthanasia. The left kidney was fixed in 4% paraformaldehyde (PFA) for hematoxylin and eosin (H&E) staining, Periodic Acid-Schiff (PAS) staining, terminal deoxynucleotidyl transferase dUTP nick end labelling (TUNEL) assay, and immunohistochemical staining, while the right kidney was kept at -80°C for western blotting.

### 2.2. Kidney Histology

After fixation in PFA for 24 h, kidney samples were dehydrated in gradient ethanol, then embedded in paraffin and sectioned (5 *μ*m) for H&E and PAS staining, which was performed as described by Chen et al. [20]. Ten fields of view were randomly chosen and observed at an original magnification of ×400. The significance of tubular injury was determined via evaluating tubular dilation, casts, vacuolization, degeneration of tubule epithelial cells, and the disappearance of the brush border [21, 22]. Histological evaluations were conducted blindly by two pathologists. The width of the nephrogenic zone was determined by averaging the width of the renal cortex containing immature glomeruli in five separate areas per kidney [23]. To identify tubular dilation, bright-field images of H&E-stained slides were analyzed and the diameter of tubular lumen was measured using ImageJ 1.51 (National Institutes of Health, Bethesda, MD, USA). Briefly, a grid of dots, 13 *μ*m apart, was superimposed on images. No dilation was defined as no dots within lumens, and tubular dilation was defined as more than one dot within each tubular lumen [24]. Tubular cell number was counted automatically using ImageJ 1.51 as per the protocol described by Parlee et al. [25].

### 2.3. Apoptosis Assay

Cell apoptosis was evaluated by TUNEL assay using an ApopTag Plus peroxidase *in situ* apoptosis detection kit (Intergen, Norcross, GA, USA). After fixation in PFA for 24 h, kidney samples were dehydrated in gradient ethanol, then embedded in paraffin and sectioned (5 *μ*m). Sections were deparaffinized in xylene and rehydrated by incubation in graded ethanols and then immersed in phosphate-buffered saline (PBS). For permeabilization, 50 *μ*L of 0.1% Triton X-100 (prepared with 0.1% sodium citrate) was added and incubated at room temperature for 8 min. Fifty microliters of a TUNEL reaction solution (prepared with enzyme and label Solutions at a ratio of 1 : 9) was added and incubated at 37°C for 30 min. Fifty microliters of converter-POD (peroxidase) was added and incubated for 30 min. Fifty microliters of 3,3′-diaminobenzidine (DAB) substrate was added for color development, and slides were washed with PBS three times. Hematoxylin was added to counterstain slides for 3 min. For derivatization, slides were immersed in 1% hydrogen chloride-ethanol solution for 3 s and then rinsed in running water for 20 min. Slides were dehydrated in gradient ethanols, immersed in xylene, then mounted with neutral balsam. TUNEL positive cells were defined as cells with darkly stained nuclei or pyknotic nuclei with apoptotic bodies. Ten fields of view from the renal cortex and medulla were randomly chosen and observed at an original magnification of ×400. TUNEL positive cell numbers from the proximal tubules and collecting ducts were counted automatically using ImageJ 1.51 as per the protocol described by Parlee et al. [25].

### 2.4. Immunohistochemical Staining

Rat kidney tissues were fixed in formalin, embedded in paraffin, sectioned at a thickness of 5 *μ*m, dewaxed, rehydrated, placed in sodium citrate, and microwaved for antigen retrieval. A nonspecific stain blocking agent was added to slides, which were then incubated for 30 min at room temperature. Anti-rat HIF-1*α* primary antibody (Thermo Fisher, Shanghai, China; dilution 1 : 200) or anti-rat catalase primary antibody (Thermo Fisher; dilution 1: 00) was added and slides incubated overnight at 4°C. After washing in PBS, the slides were incubated with secondary antibody (Gene Tech, Shanghai, China; dilution 1 : 100) for 30 min at room temperature. Excess secondary antibody was washed off. Slides were incubated with streptavidin-avidin-biotin complex, developed with 3,3′-diaminobenzidine, washed with running water, counterstained with hematoxylin, dehydrated with gradient alcohol, dried, and mounted with neutral balsam. Tissue morphology was observed and photographed under the microscope and compared with known positive staining slides. The results were interpreted blindly by two pathologists. Ten high-power fields of view (original magnification ×400) were randomly selected from each slide and images obtained with a light microscope, with 200 cells observed in each field of view. Cells with cytoplasm or nucleus stained yellow or dark brown were defined as positive cells. Semiquantitative results were obtained by determining the intensity of cell staining using image-analyzing software, ImageJ 1.51. Images in 8-bit format were used and adjusted with the Image/Adjust/Brightness/Contrast command. The threshold was then adjusted with the Image/Adjust/Threshold command. The Analyze/Measure command was then required. ImageJ 1.51 will automatically display the percentage of total area that positive cells occupy [26].

### 2.5. Western Blotting

An appropriate volume of RIPA buffer was added to tissue samples to make cell lysates. After centrifugation, the protein concentration of each sample supernatant was determined using a bicinchoninic acid protein concentration assay kit (Beyotime, Shanghai, China). A 5× Loading Buffer was used to dilute the protein samples, which were boiled in a water bath for 5 min. Proteins were separated by SDS-polyacrylamide gel electrophoresis and subsequently transferred to a polyvinylidene difluoride membrane (Millipore, Burlington, MA, USA). After incubation with primary (1 : 1000) and secondary antibodies (1 : 5000), respectively, an electrochemical luminescence solution (Sigma, St. Louis, MO, USA) was added for substrate luminescence. All bands were scanned with Chemi Imager 5500 V2.03 software (AIPha InnCh, Miami, FL, USA), and integrated density values were calculated by computerized image analysis system (Fluor Chen 2.0) and normalized to that of *β*-actin or GAPDH.

### 2.6. Statistical Analysis

GraphPad Prism 7 software (GraphPad Software, San Diego, CA, USA) was used for statistical analyses and plotting scattered dot as well as box and whisker graphs. The results of each assay were obtained after three repeated independent experiments and expressed as mean ± standard deviation (SD). The two groups of numerical data were compared by Student's *t*-test. One-way analysis of variance (ANOVA) and post hoc comparisons (Bonferroni test) were used to determine significant differences among multiple groups. Simple regression was used to correlate immunostaining intensity as an independent variable, with the TUNEL positive cell count as a dependent variable. A *P* value of less than 0.05 was considered to be statistically significant.

### 2.7. Research Approval

The study was approved by the Ethics Committee of the Shengjing Hospital, China Medical University (Shenyang, China).

## 3. Results

### 3.1. Neonatal Hyperoxia Dilates the Lumen and Decreases the Cell Density of Mature Proximal Tubules

**Figure 1:**
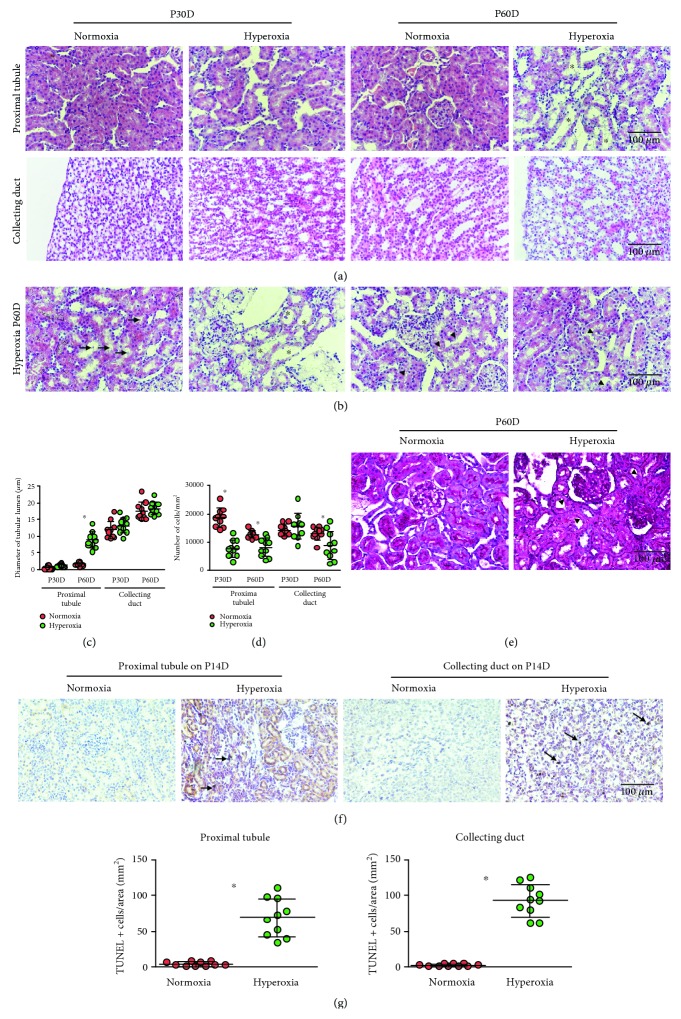
Neonatal hyperoxia dilates the lumen and decreases the cell density of mature proximal tubules. (a) The proximal tubules and collecting ducts of adult rats, including those from the 30th (P30D) and 60th postnatal days (P60D), exposed to neonatal normoxia or hyperoxia were detected by hematoxylin & eosin (H&E) staining (original magnification ×400; scale bar, 100 *μ*m). Asterisks show tubular dilation. (b) The proximal tubules from the hyperoxia group on P60D showing intratubular debris (arrows), thinner tubules (asterisks), and vacuolation (arrowheads) were detected by H&E staining (original magnification ×400; scale bar, 100 *μ*m). (c) The lumen diameters of the proximal tubules and collecting ducts from adult rats exposed to neonatal normoxia or hyperoxia are shown (mean ± standard deviation (SD)) by scatter dot plots. The error bars represent the SD of measurements for 10 rats with the mean value of 10 separate fields of view (*n* = 10). ^∗^*P* < 0.001, one-way ANOVA, Bonferroni post hoc test. (d) The cell densities of the proximal tubules and collecting ducts from adult rats exposed to neonatal normoxia or hyperoxia (mean ± SD) are shown by scatter dot plots. The error bars represent the SD of measurements for 10 rats with the mean value of 10 separate fields of view (*n* = 10). ^∗^*P* < 0.05, one-way ANOVA, Bonferroni post hoc test. (e) The proximal tubules from the hyperoxia group on P60D showing discontinuous brush border (arrowheads) were detected by Periodic Acid-Schiff (PAS) staining (original magnification ×400; scale bar, 100 *μ*m). (f) Apoptosis in the proximal tubules and collecting ducts from newborn rats exposed to normoxia or hyperoxia and harvested on the 14th postnatal day (P14D) was detected by terminal deoxynucleotidyl transferase dUTP nick end labeling (TUNEL) assay (original magnification ×400; scale bar, 100 *μ*m). Arrows indicate apoptotic bodies. (g) TUNEL positive cell numbers in mm^2^ of the proximal tubules and collecting ducts from newborn rats exposed to normoxia or hyperoxia and harvested on the 14th postnatal day are shown (mean ± SD) by scatter dot plots. The error bars represent the SD of measurements for 10 rats with the mean value of 10 separate fields of view (*n* = 10). ^∗^*P* < 0.001, Student's *t*-test.

Neonatal hyperoxia can cause bronchopulmonary dysplasia [27]. To test the hypothesis that neonatal hyperoxia may disrupt kidney development, a histologic examination was undertaken of kidney samples from the normoxia and hyperoxia groups. Morphological differences of the proximal tubules and collecting ducts from adult rats (30th and 60th postnatal days) between the normoxia and hyperoxia groups were compared by H&E staining, and tubular dilation was observed in the hyperoxia group on the 60th postnatal day (Figure 1(a)). Besides tubular dilation, intratubular debris, thinner tubules, and vacuolation in the proximal tubules were also observed in the hyperoxia group on the 60th postnatal day (Figure 1(b)). In order to facilitate the evaluation of tubular dilation, the image type was changed to an HSB stack using ImageJ 1.51 software (Supplementary [Supplementary-material supplementary-material-1]). The diameter of the proximal tubular lumen was significantly increased in the hyperoxia group (8.72 ± 2.46 *μ*m) over that of the normoxia group (1.51 ± 0.54 *μ*m) as detected on the 60th postnatal day (*P* < 0.001), while the difference in lumen diameter between the normoxia and hyperoxia groups was not significant in the collecting ducts (*P* > 0.05; Figure 1(c)). In order to quantify the number of tubular cells on the slides of H&E stains, the image type was changed to an RGB stack and the threshold adjusted using ImageJ 1.51 software according to Papadopulos et al. (Supplementary [Supplementary-material supplementary-material-1]) [28]. The number of proximal tubular cells was significantly decreased in the hyperoxia group compared to that of the normoxia group as detected on both 30th (7776 ± 3014 vs. 18605 ± 3326/mm^2^, *P* < 0.01) and 60th postnatal days (8064 ± 3213 vs. 12730 ± 1343/mm^2^, *P* < 0.001). The difference was also significant for collecting tubular cells on the 60th postnatal day (8755 ± 5012 vs. 13075 ± 2240/mm^2^, *P* < 0.05) but was not significant for the collecting ducts on the 30th postnatal day (*P* > 0.05; Figure 1(d)). In order to examine the damage of brush border, PAS was performed using sections of the proximal tubules from adult rats on the 30th and 60th postnatal days. Discontinuous brush border in the proximal tubules was observed in the hyperoxia group on the 60th postnatal day (Figure 1(e)). Since apoptosis is a critical risk for decreased cell density in the proximal tubules during nephrogenesis [29], apoptosis in tubular cells was evaluated via TUNEL on the 14th postnatal day (Figure 1(f)). The number of TUNEL positive cells per mm^2^ was significantly higher in the hyperoxia group compared to that in the normoxia group (proximal tubule: 69.20 ± 26.11/mm^2^ vs. 4.80 ± 2.97/mm^2^; collecting duct: 92.8 ± 22.40/mm^2^ vs. 3.20 ± 1.81/mm^2^, *P* < 0.001 for both; Figure 1(g)). These findings demonstrated that neonatal hyperoxia impaired proximal tubular development.

### 3.2. Hyperoxia Accelerates Attenuation of the Nephrogenic Zone in Newborn Rat Kidney

**Figure 2:**
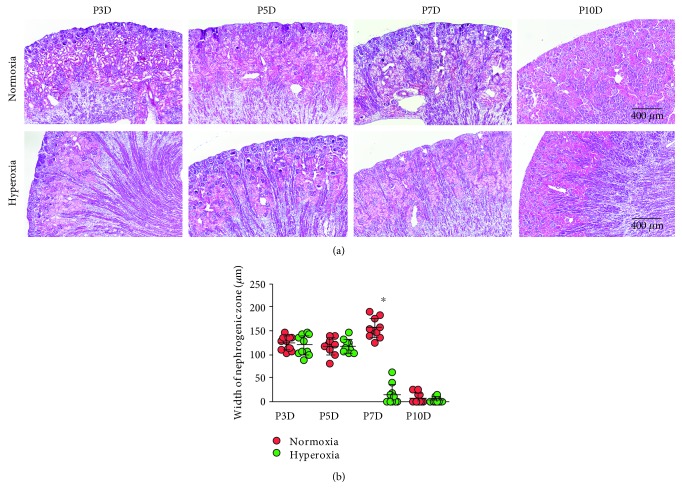
Hyperoxia accelerates the disappearance of the nephrogenic zone in newborn rat kidney. (a) The nephrogenic zone in the kidney of newborn rats, including those from the 3rd (P3D), 5th (P5D), 7th (P7D), and 10th (P10D) postnatal days after exposure to normoxia or hyperoxia and visualized by hematoxylin & eosin (H&E) staining (original magnification ×100; scale bar, 400 *μ*m). Arrows indicate the width of the nephrogenic zone. (b) The width of the neonatal nephrogenic zone, when rats were exposed to normoxia or hyperoxia, is shown (mean ± standard deviation (SD)) by scatter dot plots. The error bars represent the SD of measurements for 10 rats with the mean value of 10 separate fields of view (*n* = 10). ^∗^*P* < 0.001, one-way ANOVA, Bonferroni post hoc test.

The activity of the nephrogenic zone is critical for nephrogenesis [30]. Since neonatal hyperoxia disrupted tubular development as observed above, it is important to evaluate the effect of hyperoxia on the nephrogenic zone. The width of the nephrogenic zone in neonatal rats (3rd, 5th, 7th, and 10th postnatal days) was evaluated by H&E staining. The nephrogenic zone completely vanished on the 10th postnatal day in both the normoxia and hyperoxia groups (Figure 2(a)). In order to facilitate measuring the width of the nephrogenic zone, the image contrast was enhanced to 80% saturated pixels using ImageJ 1.51 software (Supplementary [Supplementary-material supplementary-material-1]). It was found that the width of the nephrogenic zone was significantly decreased in the hyperoxia group (14.35 ± 20.81 *μ*m) compared to that in the normoxia group (155.74 ± 21.86 *μ*m) on the 7th postnatal day (*P* < 0.001; Figure 2(b)). This demonstrated that hyperoxia accelerated attenuation of the nephrogenic zone in the newborn rat kidney.

### 3.3. MAPK/ERK Signaling Activity Is Altered in the Kidney of Newborn Rats following Hyperoxia

**Figure 3:**
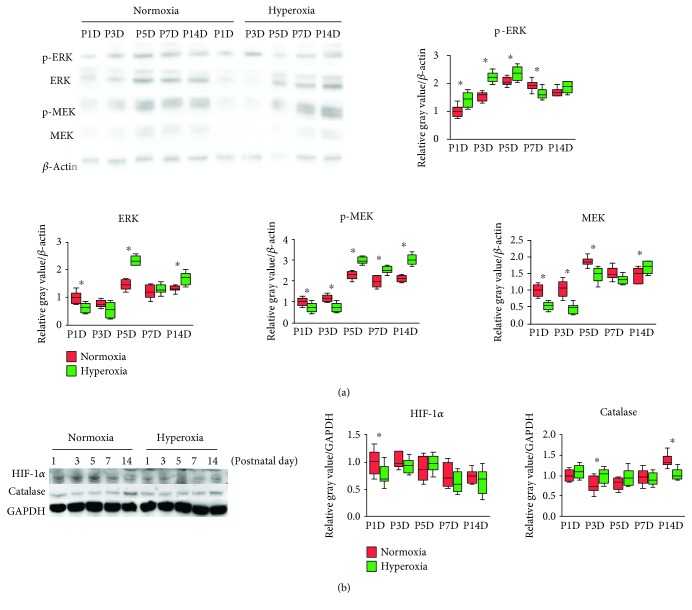
ERK/MEK signaling pathway is inactivated and then activated following hyperoxia. (a) Expression bands of phospho-ERK (p-ERK), ERK, phospho-ERK (p-MEK), and MEK in the kidneys of newborn rats exposed to normoxia or hyperoxia as detected by western blotting. Relative expression adjusted to the reference gene *β*-actin and then standardized to the value of the normoxia group on P1D is shown by box and whisker plots. The whiskers represent the minimal or the maximal gray value, and the boxes span the interquartile range of measurements for 10 rats with the mean value of 3 replicates (*n* = 10). ^∗^*P* < 0.05, one-way ANOVA, Bonferroni post hoc test. (b) Expression bands of hypoxia-inducible factor 1*α* (HIF-1*α*) and catalase in the kidneys of newborn rats exposed to normoxia or hyperoxia and detected by western blotting. Relative expression adjusted to the reference gene GAPDH and then standardized to the value of the normoxia group on P1D is shown by box and whisker plots. The whiskers represent the minimal or the maximal gray value, and the boxes span the interquartile range of measurements for 10 rats with the mean value of 3 replicates (*n* = 10). ^∗^*P* < 0.05, one-way ANOVA, Bonferroni post hoc test.

MAPK/ERK signaling is an essential pathway activated during kidney development [18]. To evaluate MAPK/ERK signaling activity following hyperoxia, the expression of phospho-ERK (p-ERK), ERK, phospho-ERK (p-MEK), and MEK in the kidneys of newborn rats was detected by western blotting (Figure 3(a)). In the normoxia group, MAPK/ERK signaling activity reached its peak on the 5th postnatal day (Figure 3(a)). In the hyperoxia group, MAPK/ERK signaling activity was mainly downregulated on the 1st and 3rd postnatal days, while it was significantly upregulated after the 5th postnatal day (*P* < 0.05; Figure 3(a)). HIF-1*α* and catalase are important downstream proteins of MAPK/ERK signaling [31–33]. Therefore, the expression of HIF-1*α* and catalase in the kidneys of newborn rats following hyperoxia was measured by western blotting (Figure 3(b)). The expression of HIF-1*α* was significantly decreased following hyperoxia on the 1st postnatal day (normoxia group 1.0 ± 0.22 vs. hyperoxia group 0.77 ± 0.18; *P* < 0.05; Figure 3(b)). With regard to catalase, its expression was significantly increased on the 3rd postnatal day following hyperoxia (normoxia group 0.78 ± 0.19 vs. hyperoxia group 1.01 ± 0.17; *P* < 0.05), while it was decreased on the 14th postnatal day (normoxia group 1.37 ± 0.16 vs. hyperoxia group 1.03 ± 0.13; *P* < 0.001; Figure 3(b)). The above observations indicated that MAPK/ERK signaling activity was originally downregulated and then upregulated in the kidney of newborn rats following hyperoxia, which was not compliant with the expression pattern of HIF-1*α* or catalase.

### 3.4. Peak Expression of HIF-1*α* and Catalase Was Reduced by Hyperoxia in Proximal Tubules

**Figure 4:**
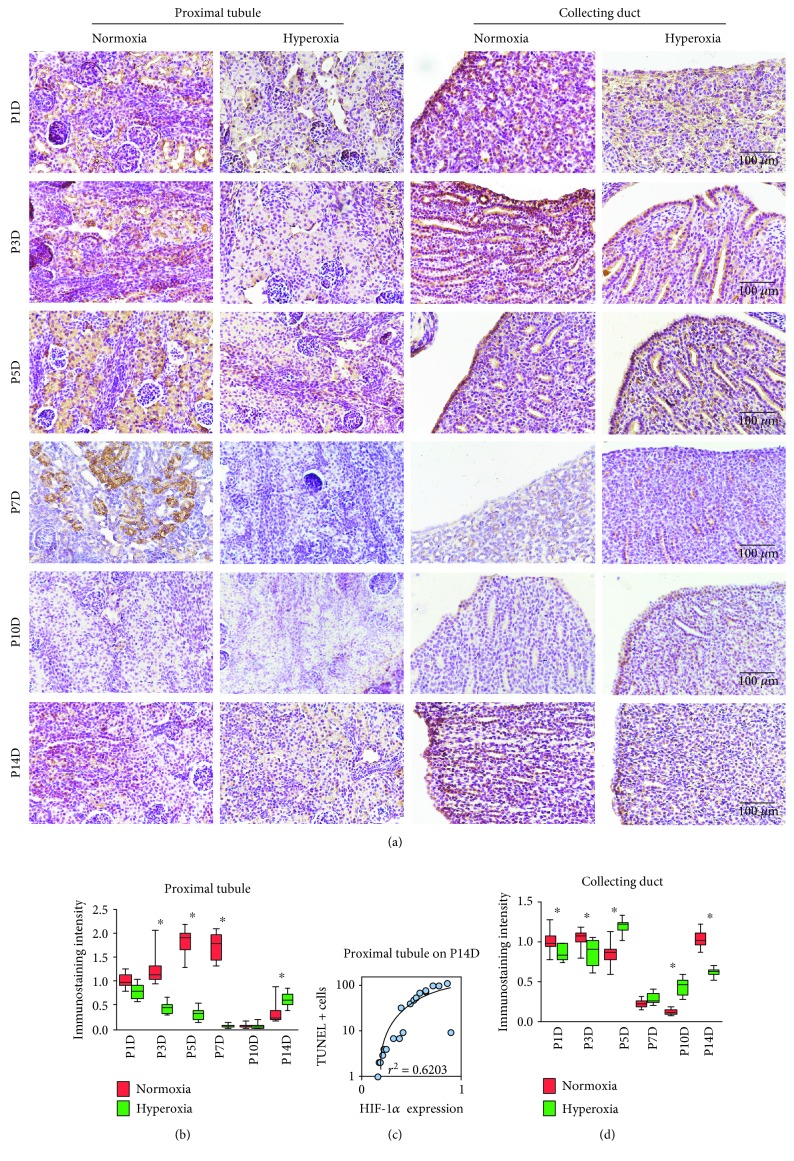
Hyperoxia downregulates hypoxia-inducible factor 1*α* (HIF-1*α*) expression in the proximal tubules and collecting ducts. (a) HIF-1*α* expression in the proximal tubules and collecting ducts of newborn rats exposed to normoxia or hyperoxia was detected by immunohistochemical staining (original magnification ×400; scale bar, 100 *μ*m). (b) The immunostaining intensity of HIF-1*α* expression in the proximal tubules from newborn rats exposed to normoxia or hyperoxia is shown by box and whisker plots. Relative expression is standardized to the value of the normoxia group on P1D. The whiskers represent the minimal intensity or the maximal intensity, and the boxes span the interquartile range of measurements for 10 rats with the mean value of 10 separate fields of view (*n* = 10). ^∗^*P* < 0.01, one-way ANOVA, Bonferroni post hoc test. (c) Linear correlation between relative immunostaining intensity of HIF-1*α* and TUNEL positive cell number per mm^2^ in the proximal tubules from newborn rats exposed to hyperoxia or normoxia and harvested on the 14th postnatal day. The graph was plotted using a log-10 scale for just the *Y* axis. The correlation coefficient (*r*^2^ = 0.6203) was found to be highly significant as shown in the graph (*n* = 20, *P* < 0.001). Simple regression. (d) The immunostaining intensity of HIF-1*α* expression in the collecting duct from newborn rats exposed to normoxia or hyperoxia is shown by box and whisker plots. Relative expression is standardized to the value of the normoxia group on P1D. The whiskers represent the minimal intensity or the maximal intensity, and the boxes span the interquartile range of measurements for 10 rats with the mean value of 10 separate fields of view (*n* = 10). ^∗^*P* < 0.05, one-way ANOVA, Bonferroni post hoc test.

To further investigate the expression of HIF-1*α* and catalase in the proximal tubules and collecting ducts following hyperoxia, immunohistochemical staining was performed on kidney tissues from neonatal rats exposed to hyperoxia or normoxia. Cytoplasmic staining of HIF-1*α* was observed in the proximal tubules and collecting ducts of neonatal rats (Figure 4(a)). In the normoxia group, HIF-1*α* expression in the proximal tubules reached its peak on the 5th postnatal day (Figure 4(b)), which was consistent with observations on MAPK/ERK signaling activity. HIF-1*α* expression in the proximal tubules was significantly downregulated following hyperoxia on the 3rd (normoxia group 1.24 ± 0.35 vs. hyperoxia group 0.44 ± 0.11, *P* < 0.001), 5th (normoxia group 1.83 ± 0.27 vs. hyperoxia group 0.32 ± 0.13; *P* < 0.001), and 7th postnatal days (normoxia group 1.73 ± 0.28 vs. hyperoxia group 0.06 ± 0.04; *P* < 0.001), showing that the expression peak of HIF-1*α* was decreased by hyperoxia in the proximal tubules. However, HIF-1*α* expression in the proximal tubules was significantly upregulated following hyperoxia on the 14th postnatal day (normoxia group 0.32 ± 0.22 vs. hyperoxia group 0.62 ± 0.14; *P* < 0.01; Figure 4(b)). A positive TUNEL cell count positively correlated with the immunostaining intensity of HIF-1*α* in the proximal tubules detected on the 14th postnatal day (*r*^2^ = 0.6203, *P* < 0.001; Figure 4(c)). HIF-1*α* expression in the collecting ducts was significantly downregulated following hyperoxia on the 1st (normoxia group 1.0 ± 0.13 vs. hyperoxia group 0.86 ± 0.10, *P* < 0.05), 3rd (normoxia group 1.04 ± 0.11 vs. hyperoxia group 0.86 ± 0.17, *P* < 0.001), and 14th postnatal days (normoxia group 1.03 ± 0.11 vs. hyperoxia group 0.62 ± 0.05, *P* < 0.001), while it was significantly upregulated following hyperoxia on the 5th (normoxia group 0.85 ± 0.15 vs. hyperoxia group 1.19 ± 0.09, *P* < 0.001) and 10th postnatal days (normoxia group 0.12 ± 0.03 vs. hyperoxia group 0.44 ± 0.10, *P* < 0.001; Figure 4(d)). No correlation between a positive TUNEL cell count and the immunostaining intensity of HIF-1*α* was found in the collecting ducts (*P* > 0.05, data not shown).

**Figure 5:**
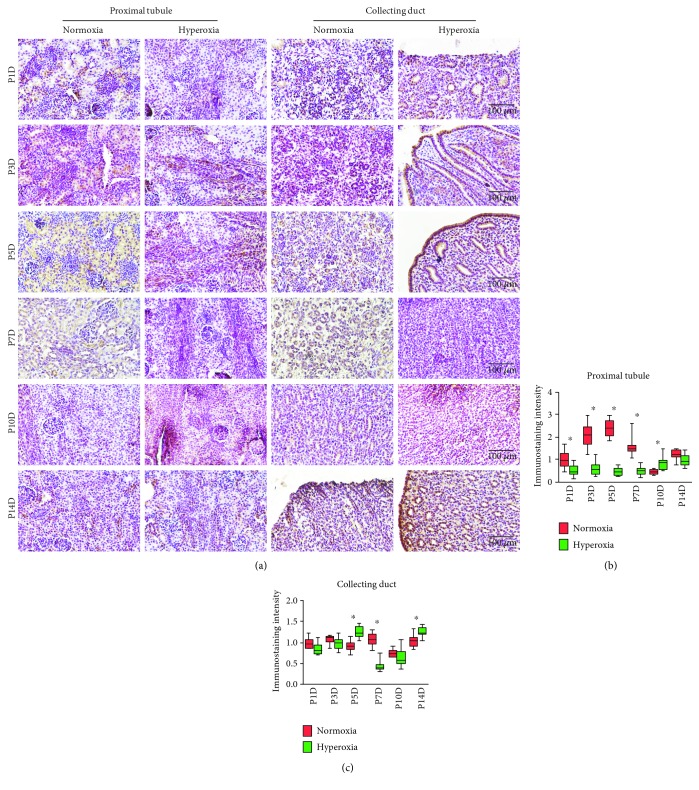
Hyperoxia downregulates catalase expression in the proximal tubules and collecting ducts. (a) Catalase expression in the proximal tubules and collecting ducts of newborn rats exposed to normoxia or hyperoxia was detected by immunohistochemical staining (original magnification ×400; scale bar, 100 *μ*m). (b) The immunostaining intensity of catalase expression in the proximal tubules exposed to normoxia or hyperoxia is shown by box and whisker plots. Relative expression is standardized to the value of the normoxia group on P1D. The whiskers represent the minimal intensity or the maximal intensity, and the boxes span the interquartile range of measurements for 10 rats with the mean value of 10 separate fields of view (*n* = 10). ^∗^*P* < 0.05, one-way ANOVA, Bonferroni post hoc test. (c) The immunostaining intensity of catalase expression in the collecting ducts exposed to normoxia or hyperoxia is shown by box and whisker plots. Relative expression is standardized to the value of normoxia group on P1D. The whiskers represent the minimal intensity or the maximal intensity, and the boxes span the interquartile range of measurements for 10 rats with the mean value of 10 separate fields of view (*n* = 10). ^∗^*P* < 0.01, one-way ANOVA, Bonferroni post hoc test.

Cytoplasmic and nucleic staining of catalase was observed in the proximal tubules and collecting ducts of neonatal rats (Figure 5(a)). In the normoxia group, catalase expression in the proximal tubules reached its peak on the 5th postnatal day (Figure 5(b)), which resembled observations on HIF-1*α* expression and MAPK/ERK signaling activity. Catalase expression in the proximal tubules was significantly downregulated following hyperoxia on the 1st (normoxia group 1.0 ± 0.39 vs. hyperoxia group 0.51 ± 0.28, *P* < 0.01), 3rd (normoxia group 2.08 ± 0.53 vs. hyperoxia group 0.60 ± 0.29, *P* < 0.001), 5th (normoxia group 2.36 ± 0.38 vs. hyperoxia group 0.46 ± 0.18, *P* < 0.001), and 7th postnatal days (normoxia group 1.56 ± 0.40 vs. hyperoxia group 0.50 ± 0.19, *P* < 0.001), showing that peak expression of catalase was attenuated by hyperoxia in the proximal tubules. However, catalase expression in the proximal tubules was significantly upregulated following hyperoxia on the 10th postnatal day (normoxia group 0.45 ± 0.10 vs. hyperoxia group 0.84 ± 0.31; *P* < 0.05; Figure 5(b)). No correlation between a positive TUNEL cell count and the immunostaining intensity of catalase was found in the proximal tubules (*P* > 0.05, data not shown). Catalase expression in the collecting ducts was significantly downregulated following hyperoxia on the 7th postnatal day (normoxia group 1.07 ± 0.15 vs. hyperoxia group 0.44 ± 0.13, *P* < 0.001), while it was significantly upregulated following hyperoxia on the 5th (normoxia group 0.91 ± 0.13 vs. hyperoxia group 1.25 ± 0.14, *P* < 0.001) and 14th postnatal days (normoxia group 1.05 ± 0.14 vs. hyperoxia group 1.26 ± 0.11, *P* < 0.01; Figure 5(c)). No correlation between a positive TUNEL cell count and the immunostaining intensity of catalase was found in the collecting ducts (*P* > 0.05, data not shown). Therefore, the expression of HIF-1*α* and catalase was downregulated by hyperoxia in the proximal tubules.

### 3.5. Hyperoxia Downregulated Expression of HIF-1*α* and Catalase in S-Shaped Bodies

**Figure 6:**
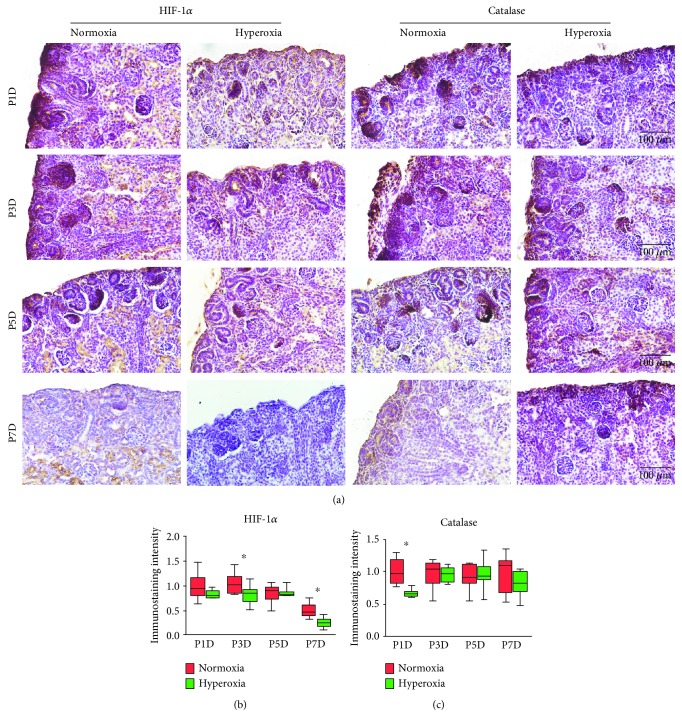
Hyperoxia alters HIF-1*α* and catalase expression in S-shaped bodies. (a) Hypoxia-inducible factor 1*α* (HIF-1*α*) and catalase expression in S-shaped bodies of newborn rats exposed to normoxia or hyperoxia was detected by immunohistochemical staining (original magnification ×400; scale bar, 100 *μ*m). (b) The immunostaining intensity of HIF-1*α* expression in S-shaped bodies exposed to normoxia or hyperoxia is shown by box and whisker plots. Relative expression is standardized to the value of the normoxia group on P1D. The whiskers represent the minimal intensity or the maximal intensity, and the boxes span the interquartile range of measurements for 10 rats and the mean value of 10 separate fields of view (*n* = 10). ^∗^*P* < 0.05, one-way ANOVA, Bonferroni post hoc test. (c) The immunostaining intensity of catalase expression in S-shaped bodies exposed to normoxia or hyperoxia is shown by box and whisker plots. Relative expression is standardized to the value of the normoxia group on P1D. The whiskers represent the minimal intensity or the maximal intensity, and the boxes span the interquartile range of measurements for 10 rats and the mean value of 10 separate fields of view (*n* = 10). ^∗^*P* < 0.001, one-way ANOVA, Bonferroni post hoc test.

The distal portion of an S-shaped body develops into a proximal tubule [34]. Since hyperoxia downregulated the expression of HIF-1*α* and catalase in the proximal tubules, whether this downregulation started from an S-shaped body stage needed to be clarified. Cytoplasmic and nucleic staining of HIF-1*α* and catalase was observed in the S-shaped bodies of neonatal rats by immunohistochemical staining (Figure 6(a)). HIF-1*α* expression in S-shaped bodies was significantly downregulated following hyperoxia on the 3rd (normoxia group 1.04 ± 0.20 vs. hyperoxia group 0.82 ± 0.18, *P* < 0.05) and 7th postnatal days (normoxia group 0.51 ± 0.14 vs. hyperoxia group 0.25 ± 0.10, *P* < 0.01; Figure 6(b)), while catalase expression in S-shaped bodies was significantly downregulated following hyperoxia on the 1st postnatal day (normoxia group 1.0 ± 0.19 vs. hyperoxia group 0.66 ± 0.06, *P* < 0.001; Figure 6(c)). The observation above demonstrated that the downregulation of HIF-1*α* and catalase following hyperoxia started at an S-shaped body stage.

### 3.6. Neonatal Hyperoxia Downregulates HIF-1*α* and Catalase Expression in Mature Proximal Tubules

**Figure 7:**
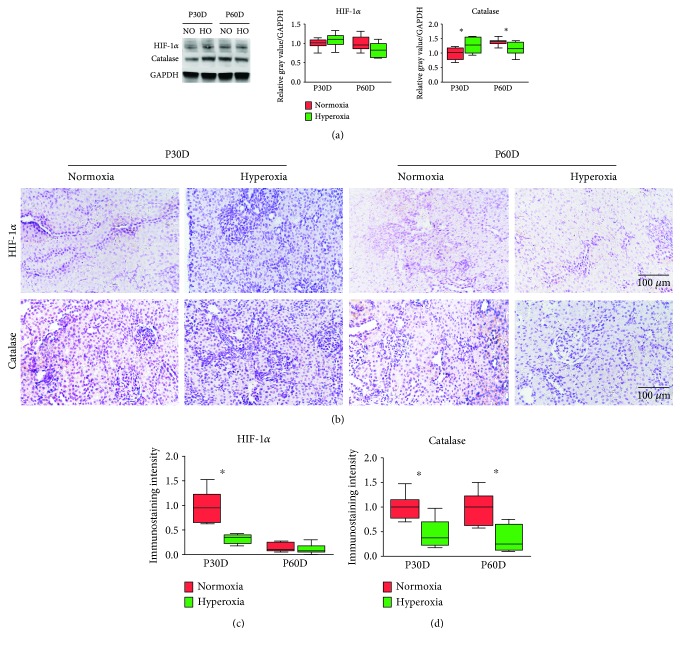
Neonatal hyperoxia downregulates hypoxia-inducible factor 1*α* (HIF-1*α*) and catalase expression in mature proximal tubules. (a) Expression bands of HIF-1*α* and catalase in mature kidneys exposed to neonatal normoxia or hyperoxia as detected by western blotting. Relative expression adjusted to the reference gene GAPDH and then standardized to the value of the normoxia group on P30D is shown by box and whisker plots. The whiskers represent the minimal or the maximal gray value, and the boxes span the interquartile range of measurements for 10 rats with the mean value of 3 replicates (*n* = 10). ^∗^*P* < 0.001, one-way ANOVA, Bonferroni post hoc test. (b) HIF-1*α* and catalase expression in the proximal tubules of adult rats exposed to neonatal normoxia or hyperoxia as detected by immunohistochemical staining (original magnification ×400; scale bar, 100 *μ*m). (c) The immunostaining intensity of HIF-1*α* expression in the proximal tubules of adult rats exposed to neonatal normoxia or hyperoxia is shown by box and whisker plots. Relative expression is standardized to the value of the normoxia group on P30D. The whiskers represent the minimal intensity or the maximal intensity, and the boxes span the interquartile range of measurements for 10 rats and a mean value of 10 separate fields of view (*n* = 10). ^∗^*P* < 0.001, one-way ANOVA, Bonferroni post hoc test. (d) The immunostaining intensity of catalase expression in the proximal tubules of adult rats exposed to neonatal normoxia or hyperoxia is shown by box and whisker plots. Relative expression is standardized to the value of the normoxia group on P30D. The whiskers represent the minimal intensity or the maximal intensity, and the boxes span the interquartile range of measurements for 10 rats and a mean value of 10 separate fields of view (*n* = 10). ^∗^*P* < 0.001, one-way ANOVA, Bonferroni post hoc test.

To evaluate the long-term effect of neonatal hyperoxia on the expression of HIF-1*α* and catalase in the kidney, western blotting was performed on kidney tissues of adult rats (Figure 7(a)). The expression of HIF-1*α* was not significantly altered following neonatal hyperoxia on the 30th and 60th postnatal days (*P* > 0.05; Figure 7(a)). However, the expression of catalase was significantly upregulated following neonatal hyperoxia on the 30th postnatal day (normoxia group 1.0 ± 0.20 vs. hyperoxia group 1.28 ± 0.25, *P* < 0.01), while it was downregulated on the 60th postnatal day (normoxia group 1.38 ± 0.11 vs. hyperoxia group 1.16 ± 0.23, *P* < 0.05; Figure 7(a)). Moderate cytoplasmic staining of HIF-1*α* and catalase was observed in the proximal tubules of adult rats (Figure 7(b)). The expression of HIF-1*α* in the proximal tubules was significantly downregulated following neonatal hyperoxia on the 30th postnatal day (normoxia group 1.0 ± 0.30 vs. hyperoxia group 0.32 ± 0.09, *P* < 0.001; Figure 7(c)), while the expression of catalase in the proximal tubules was significantly downregulated following neonatal hyperoxia on the 30th (normoxia group 1.0 ± 0.25 vs. hyperoxia group 0.46 ± 0.30, *P* < 0.001) and 60th postnatal days (normoxia group 0.96 ± 0.33 vs. hyperoxia group 0.36 ± 0.26, *P* < 0.001; Figure 7(d)). The observations above indicate that neonatal hyperoxia downregulates HIF-1*α* and catalase expression in the proximal tubule of the adult rat.

## 4. Discussion

Increasing evidence has demonstrated that neonatal hyperoxia undermines renal function [11]; however, hyperoxia-induced long-term injury to the glomeruli seems to be moderate [3, 9]. It is of interest to characterize hyperoxia-induced tubular impairment that may cause a loss of renal function. We found that neonatal hyperoxia induced the early disappearance of the nephrogenic zone in the short term. In the long term, it dilated the lumen of the proximal tubules, reduced the epithelial cell density of the proximal tubules, and induced apoptosis of tubular cells, showing that neonatal hyperoxia impairs the development of the proximal tubules. The MAPK/ERK signaling pathway promotes HIF-1*α* expression and plays a vital role in proximal tubular epithelial cell growth and differentiation [18, 35, 36], while catalase as an antioxidant plays a protective role against oxygen toxicity and apoptosis via the activation of MAPK/ERK signaling in renal cells [37, 38]. Thus, it is important to know whether MAPK/ERK signaling as well as HIF-1*α* and catalase expression in renal cells is altered by neonatal hyperoxia. Our data shows that neonatal hyperoxia altered renal MAPK/ERK signaling activity and downregulated the expression of HIF-1*α* and catalase in renal cells, while HIF-1*α* expression in the proximal tubule was positively correlated with cell apoptosis on the 14th postnatal day. This indicates that the developmental impairment induced by neonatal hyperoxia in renal cells is accompanied by an alteration of MAPK/ERK signaling and the downregulation of HIF-1*α* and catalase.

The nephrogenic zone of a fetal kidney is a narrow band along the entire inner side of the renal capsule that contains a lot of progenitor cells and undergoes continuous attenuation during kidney development [39]. The width of the nephrogenic zone, which is inversely correlated to gestational age, is considered to indicate the state of ongoing nephrogenesis [40]. By dynamic observation, we found that when exposed to hyperoxia, the nephrogenic zone vanished on the 7th postnatal day, which significantly preceded the disappearance of the nephrogenic zone exposed to normoxia. The effect of hyperoxia on various types of progenitor cells seems to be controversial. Hyperoxia stimulated the proliferation and differentiation of dopaminergic progenitors during early and late embryogenesis [41]. However, it reduced the population of progenitor cells in the lung and had no effect on cell growth but increased the differentiation of hepatic progenitor cells [42, 43]. In this regard, hyperoxia causes the early differentiation of renal epithelial cells [17], which may be the reason for an earlier vanishing of the nephrogenic zone observed in the current study.

Proximal tubule dilation is a common pathological abnormality in urinary tract obstruction, acute kidney injury, and renal dysplasia [24, 44, 45]; of these, acute kidney injury is considered a hyperoxia-induced lesion [13]. We found that neonatal hyperoxia contributed to abnormal proximal tubule dilation in adult rats. Thus, we have reason to believe that hyperoxia-induced proximal tubule dilation may represent a subclinical acute kidney injury. The two major causes of acute kidney injury are ischemic and nephrotoxic damages that may induce a loss of renal function and consequent pathological alterations, such as tubular dilatation, loss of renal microvilli, apoptosis, and even necrosis [46]. Cell density in the proximal tubules indicates the developmental status of renal epithelial cells in nephrogenesis [47]. The number of proximal tubular epithelial cells in adult mice was significantly decreased following neonatal hyperoxia in the current study. Considering that hyperoxia induces the apoptosis of alveolar epithelial cells [48], we evaluated apoptosis in tubular cells. We found that hyperoxia consistently induced apoptosis of tubular cells, which may cause acute kidney injury and impaired nephrogenesis.

MAPK/ERK signaling is a critical pathway in regulating cell proliferation and is antiapoptotic [49, 50]. In regard to kidney development, MAPK/ERK signaling initially controls niche organization and nephron progenitor maintenance, after which it is needed for the progression of differentiation in nephron precursors [18]. We observed a peak in MAPK/ERK signaling on the 5th postnatal day, while exposure to hyperoxia overactivated MAPK/ERK signaling on the 1st and 3rd postnatal days and inactivated it after the 5th postnatal day. Since MAPK/ERK signaling plays an important role in the proliferation and differentiation of nephron progenitors, we postulate that the hyperoxia-induced alteration of MAPK/ERK signaling activity may impair neonatal nephrogenesis. HIF-1*α* is a basic helix-loop-helix PAS domain-containing protein and is considered as the master transcriptional regulator of the cellular and developmental response to hypoxia [51]. Hyperoxia exposure in the neonatal period results in impaired nephrogenesis via the downregulation of HIF-1*α* [10], which is consistent with our observation that neonatal hyperoxia blunted the expression peak of HIF-1*α* in the proximal tubules. A blunted expression peak in response to neonatal hyperoxia is also observed in other tissues [52]. HIF-1*α* can trigger apoptosis in renal tubular epithelial cells [53], which is consistent with our observation that proximal tubular apoptosis is positively correlated to the expression of HIF-1*α* on the 14th postnatal day. Short-term hyperoxia downregulates HIF-1*α* via modulating MAPK/ERK signaling to reduce smooth muscle proliferation in a local blood vessel [54]. Similarly, we observed that neonatal hyperoxia inhibited HIF-1*α* expression, which was accompanied by the downregulation of MAPK/ERK signaling after the 5th postnatal day. MAPK/ERK signaling activates catalase under some circumstances [33, 55]. Catalase catalyzes the decomposition of hydrogen peroxide to water and oxygen and is a very important enzyme in protecting the cell from oxidative damage by reactive oxygen species (ROS) [56]. Hyperoxia has a protective effect against kidney damage by activating catalase [57, 58]. Our study found that neonatal hyperoxia blunted the expression peak of catalase during nephrogenesis, which is consistent with observations in lung tissue [59]. In preterm rabbits, the translational/posttranslational inhibition of catalase expression is due to an inability to induce a protective increase in hyperoxic catalase activity [60]. Such translational/posttranslational inhibition may also induce the downregulation of catalase following neonatal hyperoxia in the current study. Since catalase enhances cell viability by reducing ROS-induced apoptosis [61], the hyperoxia-induced downregulation of catalase may cause tubular apoptosis, as we observed, and undermine proximal tubular development.

Proximal tubular cells differentiate from the distal domain of S-shaped bodies during nephrogenesis [24]. HIF-1*α* and catalase expression in S-shaped bodies was investigated in order to identify the initial response of proximal tubular cells to neonatal hyperoxia. The expression of HIF-1*α* and catalase in S-shaped bodies was downregulated instantly following hyperoxia on the 1st postnatal day. Since the expression of HIF-1*α* and catalase is vital in nephrogenesis [62, 63], our findings above indicate that the nephrogenic role of HIF-1*α* and catalase expression is attenuated early by hyperoxia exposure during the S-shaped body stage. The long-term effect of hyperoxia on the expression of HIF-1*α* and catalase in the proximal tubules was also investigated in the current study, which demonstrated that HIF-1*α* and catalase expression in mature proximal tubules was downregulated following neonatal hyperoxia. However, neonatal mice exposed to hyperoxia for the first 7 days of life did not show altered catalase mRNA expression in mature lung tissue [64]. Differences in the tissues studied and the duration of exposure to hyperoxia may have resulted in the discrepancy between two studies. A relevant study was not found with regard to long-term HIF-1*α* expression following neonatal hyperoxia. Since both HIF-1*α* and catalase play a protective role against oxidative stress in the proximal tubules [65, 66], our findings above indicate that the protective role of HIF-1*α* and catalase expression against hyperoxia is attenuated continuously up to adulthood.

## 5. Conclusions

In summary, our present investigation shows that neonatal hyperoxia causes an early disappearance of the nephrogenic zone and the impaired development of the proximal tubules, which is accompanied by a significant downregulation of MAPK/ERK signaling activity and the expression of HIF-1*α* and catalase throughout nephrogenesis, from S-shaped bodies to mature proximal tubules. Overall, our results indicate that neonatal hyperoxia may impair nephrogenesis by inhibiting MAPK/ERK signaling as well as HIF-1*α* and catalase expression. Therapeutic management targeting MAPK/ERK signaling, HIF-1*α*, or catalase may serve to protect against hyperoxia-induced oxidative damage in neonatal proximal tubules.

## Data Availability

The data used to support the findings of this study are available from the corresponding author upon request.

## Supplementary Materials

Supplementary Materials Supplementary figure 1: (a) to facilitate measuring the diameter of the tubular lumen, the image type was changed to a HSB stack (original magnification ×400; scale bar, 100 *μ*m). Arrows indicate the diameter of the tubular lumen. (b) To facilitate measuring the cell density, the image type was changed to an RGB stack and the threshold was adjusted (original magnification ×400; scale bar, 100 *μ*m). (c) To facilitate measuring the width of the neonatal nephrogenic zone, the contrast of the image was enhanced to 80% saturated pixels (original magnification ×100; scale bar, 400 *μ*m). Arrows indicate the width of the nephrogenic zone.
