# Supplementary figures and images for: Proximal Tubular Development Is Impaired with Downregulation of MAPK/ERK Signaling, HIF-1α, and Catalase by Hyperoxia Exposure in Neonatal Rats
Source: Oxid Med Cell Longev. 2019 Aug 28;2019:9219847. doi: 10.1155/2019/9219847 (PMC6735195; doi:10.1155/2019/9219847)

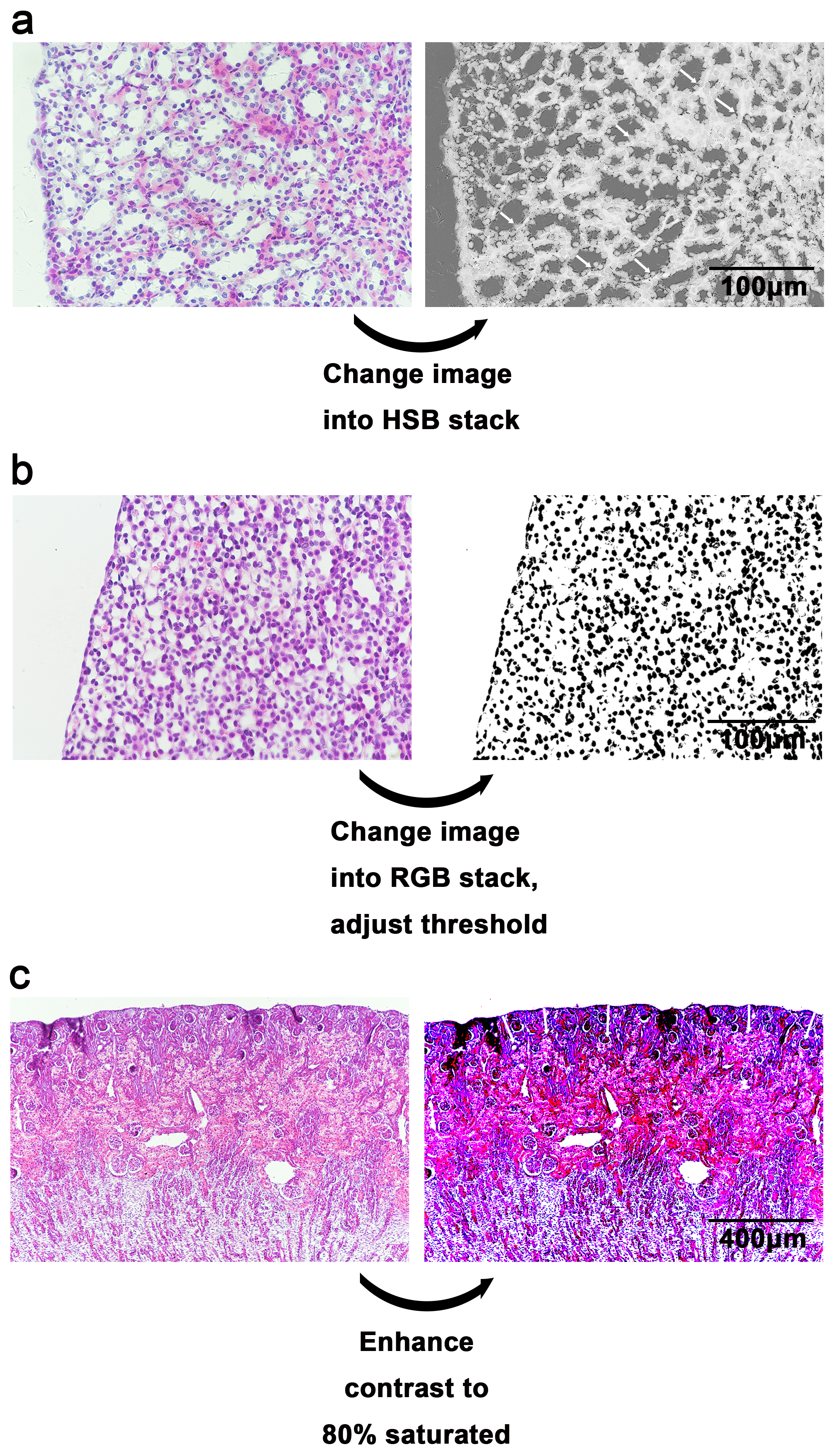

Supplement: Supplementary Materials — Supplementary figure 1: (a) to facilitate measuring the diameter of the tubular lumen, the image type was changed to a HSB stack (original magnification ×400; scale bar, 100 μm). Arrows indicate the diameter of the tubular lumen. (b) To facilitate measuring the cell density, the image type was changed to an RGB stack and the threshold was adjusted (original magnification ×400; scale bar, 100 μm). (c) To facilitate measuring the width of the neonatal nephrogenic zone, the contrast of the image was enhanced to 80% saturated pixels (original magnification ×100; scale bar, 400 μm). Arrows indicate the width of the nephrogenic zone. [file 9219847.f1.tif]
